# A Rare Case of Cervical Solitary Fibrous Tumor in a Pediatric Patient: Case Report and Literature Review

**DOI:** 10.3390/neurosci6020049

**Published:** 2025-06-01

**Authors:** Eleonora Becattini, Lorenzo Sgarbanti, Giuseppina Bevacqua, Valentina Grespi, Carlo Conti

**Affiliations:** 1 Unit of Neurosurgery, Azienda Ospedaliera S. Maria, 05100 Terni, Italy; e.becattini@aospterni.it (E.B.); giuseppina.bevacqua@aospterni.it (G.B.); c.conti@aospterni.it (C.C.); 2 Neurosurgery Unit, Department of Translational Medicine, University of Ferrara, 44121 Ferrara, Italy; lorenzo.sgarbanti@gmail.com; 3 Department of Neurosurgery, University Hospital S. Anna of Ferrara, 44124 Ferrara, Italy; 4 Stem Cell Laboratory, Cell Factory and Biobank, Azienda Ospedaliera S. Maria, 05100 Terni, Italy

**Keywords:** solitary fibrous tumors, neurosurgery, central nervous system

## Abstract

Solitary fibrous tumors (SFTs) are rare mesenchymal neoplasms of fibroblastic origin. In this study, we report a rare case of cervical SFT in a pediatric patient, a rare phenomenon since the incidence is particularly rare in pediatric patients according to the literature. Typical radiological features of the lesion may lead to misdiagnosis. Image study and immunohistochemistry are crucial for its correct diagnosis. Their imaging characteristics often resemble meningiomas or schwannomas, making differential diagnosis challenging. Immunohistochemical markers such as CD34 and STAT6 remain essential for definitive diagnosis.

## 1. Introduction

Solitary fibrous tumors (SFTs) are rare mesenchymal neoplasms of fibroblastic origin [1,2]. Initially described in 1931 by Klemperer and Rabin as pleural-based tumors, subsequent research has revealed their widespread anatomical distribution, including involvement of the central nervous system (CNS) and, more infrequently, the spinal cord. Although SFTs have been documented in numerous extrathoracic locations such as the head and neck, extremities, abdomen, pelvis, and retroperitoneum [3,4], their presence within the spinal canal, particularly at the cervical level, remains an exceedingly rare clinical entity. Spinal SFTs are more commonly found in the thoracic region, with fewer reported cases involving the cervical, lumbar, and sacral areas [3,4,5,6].

The World Health Organization (WHO) classification of CNS tumors, most recently updated in 2021, categorizes SFTs into three grades based on their histopathological characteristics, particularly mitotic activity and necrosis [7,8]. Grade I tumors are typically well-differentiated, highly collagenous, and exhibit low cellularity. Grade II lesions display increased cellularity and a more prominent vasculature, historically referred to as hemangiopericytomas (HPCs). Grade III SFTs, formerly known as anaplastic HPCs, are markedly cellular and exhibit a higher likelihood of malignant transformation, recurrence, and, albeit rarely, metastasis. Despite their overall indolent behavior, SFTs can demonstrate an unpredictable clinical course, necessitating a thorough diagnostic workup and tailored therapeutic strategies. In the 2007 version of the WHO classification, SFTs were included in the group of tumors of mesenchymal and meningothelial origin and graded as two and three. The 2016 update introduced a refine classification, recognizing SFTs as a distinct entity, solitary fibrous tumor/hemangiopericytoma, and assigning three separate grades to reflect their biological heterogeneity [9].

The clinical presentation of spinal SFTs is highly variable and depends on the tumor’s anatomical location, size, and rate of progression. The symptoms exhibited by patients may be related to spinal cord compression, including localized pain, radiculopathy, sensory deficits, motor weakness, and, in some cases, sphincter dysfunction. Less commonly, mainly in cases involving bone structure or extra spinal involvement, clinical manifestations may include spinal deformity or nonspecific back pain. Given their rarity and nonspecific symptomatology, SFTs are frequently misdiagnosed as more common spinal lesions, such as meningiomas or schwannomas. This diagnostic ambiguity highlights the critical importance of advanced neuroimaging techniques, particularly magnetic resonance imaging (MRI), and the implementation of immunohistochemical analysis for accurate tumor identification. MRI remains the gold standard for initial radiological evaluation, with schwannomas typically appearing as well-circumscribed, isointense masses on T1-weighted images and hyperintense on T2-weighted sequences, with strong contrast enhancement. However, distinguishing these tumors from meningiomas or schwannomas based on imaging alone is challenging. Histopathological and immunohistochemical evaluation play a critical role in definitive diagnosis, with SFTs characteristically exhibiting a strong and diffuse expression of CD34 and nuclear positivity for STAT6. These markers help to differentiate SFTs from meningiomas, which typically express epithelial membrane antigen (EMA), and schwannomas, which are positive for S100 and SOX10.

Given the rarity of spinal SFTs, there is no consensus on optimal treatment strategies, although surgical resection remains the mainstay of therapy, with complete excision being the most important prognostic factor. The extent of resection significantly impacts recurrence rates, and in cases of subtotal resection, the addition of radiotherapy to reduce the likelihood of tumor regrowth is an option, although the effectiveness of this approach in improving overall survival remains controversial. Long-term surveillance with periodic MRI is essential, given the potential for late recurrence or malignant transformation, particularly in higher-grade SFTs. This study presents a rare case of a pediatric cervical SFT, a highly uncommon entity, with a review of the literature on intradural cervico-thoracic SFTs in pediatric patients. We present detailed insights into the clinical features, diagnostic challenges, and therapeutic considerations associated with these rare neoplasms. Given the limited number of reported cases in children, this case contributes valuable data to the existing body of knowledge and underscores the need for continued investigation into the behavior and optimal management of spinal SFTs.

## 2. Case Presentation

**Figure 1:**
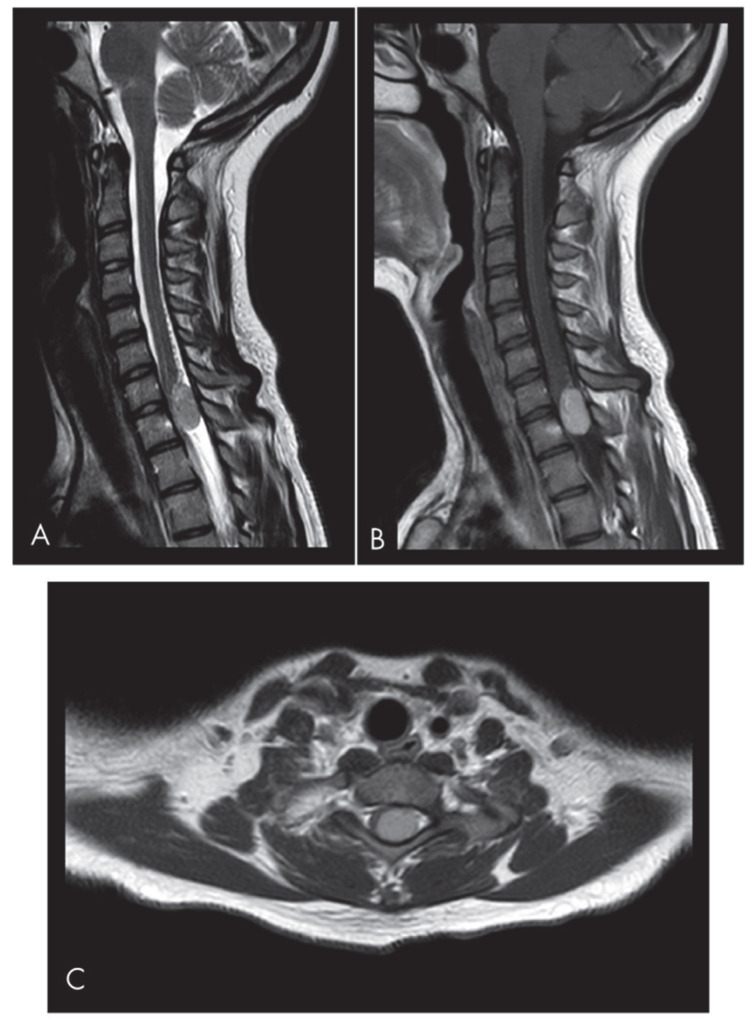
Sagittal (**A**) and axial (**C**) T2-weighted MRI images reveal an isointense lesion at the C7-T1 level causing significant spinal cord compression. The left paramedian lesion occupies the spinal canal at the C7-T1 level. The sagittal T2-weighted scan shows the inhomogeneous isointensity of the lesion and initial signs of syringomyelia. (**B**) The sagittal T1-weighted images show the enhancing mass.

**Figure 2:**
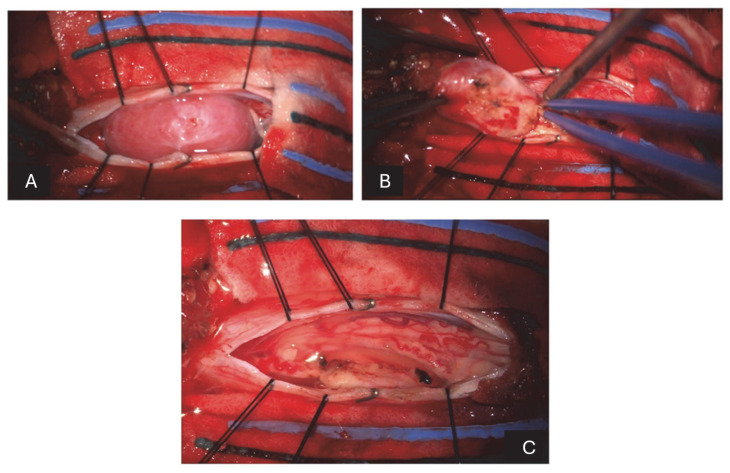
(**A**) After incising the dura mater and suspending the flaps, there is a pinkish-white lesion emerging posterior to the spinal cord. (**B**) After reducing the size of the lesion with the ultrasonic aspirator, it dissects from the adjacent nerve structures to which it is closely adhered. (**C**) After the removal of the last fragment and some washings, there is a complete resection of the lesion and release of the compressed nerve structures.

In 2023, a 15-year-old female patient was referred to the Neurosurgery Department of Santa Maria Hospital (Terni, Italy) with a progressively worsening neurological condition. The initial clinical presentation was characterized by hypoesthesia localized to the right lumbar region and the anteroinferior surface of the right lower limb. Over time, this sensory impairment was accompanied by progressive muscle weakness affecting both lower limbs, with greater severity on the left side. Additionally, the patient developed mild urinary incontinence, suggestive of early involvement of sacral autonomic pathways. An MRI of the cervical spine revealed an expansive, well-circumscribed intradural mass measuring 10 × 14 × 22 mm at the C7-T1 level, exerting significant spinal cord compression. The lesion was initially reported as a meningioma based on its radiological features also without an evident dural tail (Figure 1). Given the progressive neurological deficits and imaging findings, a multidisciplinary neurosurgical team conducted a comprehensive evaluation, concluding that surgical intervention was warranted. The patient subsequently underwent a C7-T1 laminectomy via a posterior midline approach (Figure 2). A gross total resection of the tumor was successfully performed under continuous electrophysiological monitoring, including motor and somatosensory evoked potentials, which remained stable throughout the procedure. The postoperative course was uneventful, with significant improvement in preoperative symptoms. No new neurological deficits were observed. She was mobilized on the first postoperative day and discharged on postoperative day five in a good general condition.

Histopathological examination confirmed the diagnosis of a solitary fibrous tumor (SFT). Immunohistochemical analysis demonstrated strong positivity for CD34 and STAT6, supporting the diagnosis. In contrast, markers such as S100, SOX10, EMA, CD99, OLIG2, and GFAP were negative, thereby excluding alternative diagnostic possibilities such as schwannoma, meningioma, and glial neoplasms. The Ki-67 proliferation index was approximately 10%, indicative of a moderate proliferative potential.

**Figure 3:**
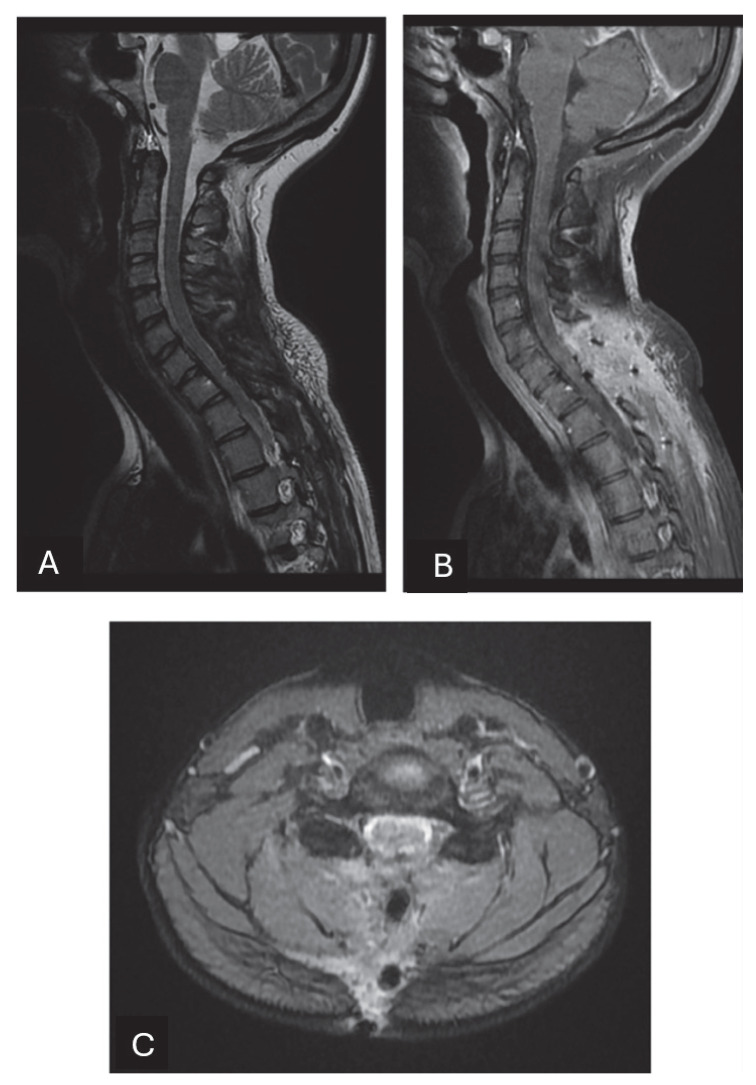
In this MRI executed 3 months after the surgical resection, complete resection of the tumor is confirmed. The spinal cord surfaces are released from the marked compression of the tumor. (**A**) Sagittal T2-weighted images show the absence of signs of syringomyelia. The lamina of the seventh cervical vertebra and the first dorsal vertebra were removed and the scar tissue can be noticed. There are no alterations in the physiological curvature of the spine. Sagittal (**B**) and axial (**C**) T1-weighted post-contrast images after injection show no signs of residual or recurrent disease.

Given the histological findings, the patient was referred for an oncohematological consultation and was enrolled in a structured clinical and instrumental follow-up program. Genetic counseling was also recommended to assess potential hereditary predispositions. A follow-up cervical spinal MRI, performed three months postoperatively, confirmed the complete resection of the lesion, with no evidence of intradural enhancement suggestive of residual or recurrent tumor tissue (Figure 3). Given the achievement of negative surgical margins and the absence of histopathological features suggestive of malignancy, adjuvant radiotherapy was not indicated. The patient remains under close surveillance, with the next scheduled clinical and radiological follow-up planned for seven months post-surgery.

## 3. Discussion

Solitary fibrous tumors (SFTs), first identified by Klemperer and Rabin in 1931 as pleural-based mesenchymal neoplasms, are now recognized as fibroblastic tumors with a widespread anatomical distribution. The WHO’s 2021 classification divides SFTs into three grades based on cellularity, collagen content, and vascular features. While typically benign, high-risk features such as elevated Ki-67 index and tumor size >10 cm^3^ are associated with recurrence, metastasis, and, rarely, disease-related mortality [1,10].

The current report presents an exceptionally rare case of a cervical SFT in a pediatric patient. SFTs involving the CNS are uncommon, and those located in the spinal canal represent a minority of cases. According to our review of the literature, only eight cases—including the present one—of intradural cervico-thoracic SFTs in pediatric patients have been described (Table 1). Considering the latest update of WHO classification, we included tumors classified as “hemangiopericytoma” in the article published before 2021.

Among the included cases, five were female and three were male, with a mean age of 14.1 years (range 10–17 years). As summarized in Table 1, the review included two patients with cervical lesions, three with thoracic lesions, and three with junctional lesions (one cervico-thoracic and two thoraco-lumbar). Among the patients with thoracic lesions, one presented with a double intradural lesion at two adjacent levels and another presented with a recurrent tumor. Six patients out of eight presented with an extramedullary lesion and two with an intramedullary tumor. Regarding clinical presentation, motor deficits were the onset symptom in six cases, hypoesthesia/hyperreflexia were present in two cases, and urinary incontinence/cauda equina syndrome was shown in three cases. One patient presented with spinal deformity (scoliosis). All the patients included in our review underwent surgical treatment with gross total resection and none demonstrated recurrence during follow-up. Data about adjuvant treatment were included in six cases out of eight, and only three of them underwent postoperative radiotherapy. No recurrence was reported and the mean follow up time was 24.3 months (range 1–60 months).

Considering the low incidence rate of SFTs, particularly in pediatric patients, and the fact that the literature on spinal SFT is limited to case reports and small case series, definitive conclusions regarding epidemiology, treatment protocols, and long-term prognosis remain elusive.

According to the existing literature, the vast majority of SFT cases occur in adults (up to 90–94% of patients) with the average age at primary diagnosis ranging from 32 to 41 years [14,15].

Moreover, a slight male predominance has been observed in [13,15]. A review on spinal SFTs published in 2017 by Albert et al. reported a male vs. female predominance of 56.4% vs. 43.6% [15].

As with other spinal neoplasms, the most common localization for spinal SFT is the thoracic spine. The intradural, and more specifically the extramedullary compartment of the spine is the most common space where spinal SFT can be found, although many cases of extradural localization have been described, often with extension into the epidural space [15].

The clinical presentation of SFTs varied according to the specific anatomic site and lesion size. In case of intradural localization, the most common symptoms and neurological signs are hypo-hyperalgesia, radicular pain, hypo-hyperreflexia, motor deficit, and limb weakness. In rare cases, sphincter dysfunction occurs. When it is associated with hypoglycemia and high levels of IGF-1, it is possible to diagnose Doege–Potter syndrome, excluding alternative causes [2,17]. The SFT reported in this case was located at the C7-T1 level, and the patient subsequently presented with hyposthenia in the lower limbs and mild urinary incontinence.

Surgery plays a crucial role in the management of this tumor because it is the only potentially curative treatment for SFT. The goal of surgery is total excision with free resection margins, wherever possible, because the extent of resection strongly influences prognosis. Few studies report a clear indication for postoperative RT after GTR with strong evidence of improvement in local control and overall survival [16]. In the case of incomplete resection, postoperative radiotherapy, either as gamma-knife radiosurgery or external beam radiation therapy, is recommended to reduce recurrence, but was not shown to improve the overall survival [18]. After complete resection, no adjuvant radiotherapy or chemotherapy is needed [1,19]. In metastatic cases of SFTs, radical surgery associated with postoperative radiotherapy, in some studies, was shown to be the best treatment available to achieve long-term survival [2,6], but due to the rarity of the tumor and the lack of standardized clinical trials, this is debatable. Chemotherapy is infrequently employed, and the literature offers little evidence of its efficacy [13].

Malignant transformation, recurrence, or metastasis can recur many years after initial diagnosis and after total resection. Thus, long-term follow-up with MRI is always recommended to detect any signs of malignant transformation or recurrence after surgery [20]. It may include annual or semiannual imaging for the first 5 years and every 5 years thereafter. Semiannual MRI scans are suggested for cases in which resection was partial and for those with malignant transformation [2,5]. In a literature review, Albert et al. noted that recurrence often occurs between 5 and 14.5 years following surgery, further supporting the need for long-term follow-up. Additionally, they reported three cases in which patients with prior intracranial SFTs subsequently developed spinal lesions with radiological features consistent with drop metastases [15].

Typical radiological manifestations of the lesion may lead to misdiagnosis. Image study and immunohistochemistry are crucial for its correct diagnosis. Distinguishing SFTs from other intradural spinal tumors, such as meningiomas, schwannomas, and sometimes neurofibromas, is essential for proper management.

On an MRI, considered the gold standard for diagnosis, SFTs present as a dural-based mass and appear isointense on T1-weighted sequences and hypointense on T2-weighted sequences with strong homogeneous enhancement [21,22]. The most common differential diagnoses at the CNS level include meningioma or schwannoma.

Schwannoma is more common in the lumbar region and usually presents as a heterogeneously high T2-signal dumbbell-shaped mass with cystic degeneration and marked enhancement [10]. Meningioma usually presents with an isointense T1- and T2-signal mass with characteristic dural tail signs and thickened dura [20].

However, some particular findings in SFT MR images include the involvement of adjacent bone with osseous erosion instead of hyperostosis, the hypervascular nature of the lesion on MR angiography with prominent flow-voids on T2-weighted images (*T*2WI), and the absence of intratumoral calcification [21]. In addition to MRI, a CT scan is beneficial to evaluate bone structures, especially in those cases where the tumor is located at the vertebral or paravertebral level.

Although not decisive, some intraoperative findings can suggest an SFT diagnosis. Intradural extramedullary SFTs often present with a firm attachment to the spinal cord and no clear arachnoidal interface. Furthermore, STFs, unlike neurinomas, lack involvement of the spinal roots and, unlike meningiomas, have a hard tumor consistency, little to no vascularization, and an absent or weak dural adherence [22].

The study of IHC staining is essential for the definitive diagnosis of SFTs by differentiating them from other neoplasms. SFTs typically exhibit strong, diffuse positivity for CD34 (80–100% of cases), a cell-surface glycoprotein that functions as a cell–cell adhesion factor, and often co-expression of Bcl-2 and vimentin [6].

In contrast, it has negative reactions for EMA and S-100, cytokeratin, SMA, desmin, S-100, EMA, HMB-45, and c-kit, which are usually negative [6,9,23,24]. Under histological and cytological examination, meningioma shows psammoma bodies, sinuous spindle cells, nuclear grooves, and intranuclear inclusions with abundant eosinophilic cytoplasm [9,24,25]. CD34-positive fibroblasts have been discovered on the dural basal layer, subpial interstice, perineurium, and endoneurium of normal nerves, and in the intraparenchymal perivascular connective tissue; thus, there is a tight connection between SFTs and meningiomas [2,10]. Most meningiomas are positive for the EMA antigen; in contrast, SFTs are negative [6].

## 4. Conclusions

The presented case and literature review highlight the extraordinary rarity of solitary fibrous tumors (SFTs) in the pediatric population, particularly at the cervical level of the spinal cord. The differential diagnosis of these neoplasms remains complex due to their radiological similarity to more commonly encountered spinal tumors, such as meningiomas and schwannomas. This diagnostic challenge necessitates a multidisciplinary approach that combines advanced neuroimaging and immunohistochemical characterization. The accurate identification of biomarkers, particularly CD34 and STAT6, is essential for definitive diagnosis and guiding appropriate therapeutic strategies. Surgical treatment remains the cornerstone of clinical management, with the primary goal being complete resection to minimize the risk of recurrence. Although the prognosis is generally favorable when free resection margins are achieved, the potential for recurrence or malignant transformation, albeit rare, underscores the necessity for long-term follow-up. The lack of standardized treatment protocols for spinal SFTs, due to their low incidence, highlights the importance of case sharing and a continued literature review to refine diagnostic and therapeutic strategies.

In conclusion, the management of these rare neoplasms necessitates a comprehensive clinical and radiological evaluation and sustained postoperative follow-up to optimize patient outcomes and preserve quality of life The future directions of research will be instrumental in refining prognostic criteria and optimizing the available therapeutic options. This will be achieved through the collection of data from larger case series.

## Figures and Tables

**Table 1:** Demographic and pathological characteristics of previously reported cases.

Reference	N° Cases	Sex	Age	LevelsInvolved	Vertebral Compartment	Onset Symptoms	Treatment	Recurrence	Follow-Up
Jallo, 2005 [11]	1	M	17	T5-T6	Intramedullary	Scoliosis, spastic paraparesis	GTR NA	No	19
Turk, 2015 [12]	1	F	15	T9-T10	Intramedullary	NA	GTR No RT	No	NA
Das, 2015 [13]	1	F	12	T11-L1	Intradural Extramedullary	Paraparesi	GTR RT	No	9
Kaur, 2015 [14]	1	M	16	T9	Intradural Extramedullary	Paraparesi, cauda	GTR RT	No	60
Albert, 2017 [15]	1	M	10	C1-C3	Intradural Extramedullary	Right arm weakness (4/5), hyperreflexia, Babinsky	GTR No RT	No	12
Singla, 2020 [16]	1	F	12	T11-L1	Intradural Extramedullary	Cauda syndrome	GTR RT	No	53
Koduru, 2020 [3]	1	F	16	C5-C7	Intradural Extramedullary	Right arm weakness (3/5)	GTR NA	NA	1
Present case	1	F	15	C7-T1	Intradural Extramedullary	Lower limb hypoesthesia and weakness, mild urinary incontinence	GTR No RT	No	16

GTR: gross total resection; RT: radiation therapy; NA: not available.

## Data Availability

Data from the study will be available upon reasonable request to the corresponding author.

## Abbreviations

The following abbreviations are used in this manuscript:

SFTs	Solitary fibrous tumors
CNS	Central nervous system
IHC	Immunohistochemical
